# Birth Weight in Relation to Leisure Time Physical Activity in Adolescence and Adulthood: Meta-Analysis of Results from 13 Nordic Cohorts

**DOI:** 10.1371/journal.pone.0008192

**Published:** 2009-12-16

**Authors:** Lise Geisler Andersen, Lars Ängquist, Michael Gamborg, Liisa Byberg, Calle Bengtsson, Dexter Canoy, Johan G. Eriksson, Marit Eriksson, Marjo-Riitta Järvelin, Lauren Lissner, Tom I. Nilsen, Merete Osler, Kim Overvad, Finn Rasmussen, Minna K. Salonen, Lene Schack-Nielsen, Tuija H. Tammelin, Tomi-Pekka Tuomainen, Thorkild I. A. Sørensen, Jennifer L. Baker, for the NordNet Study Group

**Affiliations:** 1 Institute of Preventive Medicine, Center for Health and Society, Copenhagen University Hospital, Copenhagen, Denmark; 2 Department of Surgical Sciences, Orthopaedics, Uppsala University, Uppsala, Sweden; 3 Uppsala Clinical Research Center, Uppsala University, Uppsala, Sweden; 4 Department of Public Health and Community Medicine, University of Gothenburg, Gothenburg, Sweden; 5 The Northwest Institute for Bio-Health Informatics, School of Community-Based Medicine, The University of Manchester, Manchester, United Kingdom; 6 Institute of Health Sciences and Biocenter Oulu, University of Oulu, Oulu, Finland; 7 Department of General Practice and Primary Health Care, University of Helsinki, Helsinki, Finland; 8 National Institute of Health and Welfare, Helsinki, Finland; 9 Helsinki University Central Hospital, Helsinki, Finland; 10 Vasa Central Hospital, Vasa, Finland; 11 Folkhälsan Research Center, Helsinki, Finland; 12 Department of Public Health Sciences, Karolinska Institutet, Stockholm, Sweden; 13 Department of Epidemiology and Public Health, Imperial College London, London, United Kingdom; 14 Department of Child and Adolescent Health, National Institute of Health and Welfare, Helsinki, Finland; 15 Human Movement Science Programme, Norwegian University of Science and Technology, Trondheim, Norway; 16 Research Centre for Prevention and Health, Glostrup University Hospital, Glostrup, Denmark; 17 Institute of Public Health, University of Copenhagen, Copenhagen, Denmark; 18 Department of Cardiology and Department of Clinical Epidemiology, Aalborg Hospital, Aarhus University Hospital, Aalborg, Denmark; 19 Diabetes Prevention Unit, Department of Health Promotion and Chronic Disease Prevention, National Institute for Health and Welfare, Helsinki, Finland; 20 Department of Human Nutrition, Faculty of Life Science, University of Copenhagen, Frederiksberg, Denmark; 21 Finnish Institute of Occupational Health, Oulu, Finland; 22 LIKES - Research Center for Sport and Health Sciences, Jyväskylä, Finland; 23 Research Institute of Public Health, University of Kuopio, Kuopio, Finland; Lerner Research Institute, United States of America

## Abstract

**Background:**

Prenatal life exposures, potentially manifested as altered birth size, may influence the later risk of major chronic diseases through direct biologic effects on disease processes, but also by modifying adult behaviors such as physical activity that may influence later disease risk.

**Methods/Principal Findings:**

We investigated the association between birth weight and leisure time physical activity (LTPA) in 43,482 adolescents and adults from 13 Nordic cohorts. Random effects meta-analyses were performed on categorical estimates from cohort-, age-, sex- and birth weight specific analyses. Birth weight showed a reverse U-shaped association with later LTPA; within the range of normal weight the association was negligible but weights below and above this range were associated with a lower probability of undertaking LTPA. Compared with the reference category (3.26–3.75 kg), the birth weight categories of 1.26–1.75, 1.76–2.25, 2.26–2.75, and 4.76–5.25 kg, had odds ratios of 0.67 (95% confidence interval: 0.47, 0.94), 0.72 (0.59, 0.88), 0.89 (0.79, 0.99), and 0.65 (0.50, 0.86), respectively. The shape and strength of the birth weight-LTPA association was virtually independent of sex, age, gestational age, educational level, concurrent body mass index, and smoking.

**Conclusions/Significance:**

The association between birth weight and undertaking LTPA is very weak within the normal birth weight range, but both low and high birth weights are associated with a lower probability of undertaking LTPA, which hence may be a mediator between prenatal influences and later disease risk.

## Introduction

It has been shown that babies born small have a higher risk of developing type 2 diabetes [1]–[3], the metabolic syndrome [4], and coronary heart disease [5], [6] later in life. Furthermore, a high birth weight is associated with later risk of obesity [7]–[10] and type 2 diabetes [1] and both low and high birth weights are associated with a higher risk of all-cause mortality in adulthood [11]. These associations have been interpreted as a consequence of non-optimal growth during foetal life and infancy with long-term effects on cardiovascular function, referred to as the “developmental origins of disease hypothesis” [3]. It has been suggested that not only physiological outcomes directly related to the later disease processes, but also behavior may be determined early in life [12]. Such associations could contribute to explaining the relationship between birth weight and adult disease and lead towards an understanding of the underlying mechanisms as well as have public health implications.

There is strong evidence that physical inactivity is a major predictor for many chronic diseases and is associated with all-cause mortality [13], [14]. Birth weight is positively associated with adolescent and adult lean body mass [15], [16] and muscle strength [15], which could, in turn, lead to a direct association between birth weight and an active lifestyle. On the other hand, high birth weight is also associated with an increased risk of obesity in later life [7]–[10], which might have the opposite effect. If leisure time physical activity (LTPA) is associated with birth weight, this association has the potential of mediating the effect of birth weight on the later risk of disease by a modifiable risk factor. Previous research on the relationship between birth weight and level of physical activity in children has not identified any associations [12], [17], nor have consistent ones been identified in adults [4], [18]–[20]. These investigations assumed a linear association across the range of birth weights, and none explicitly investigated the shape of the association.

The aim of the present study was to explore the nature of the association between birth weight and LTPA later in life in the context of a Nordic collaboration that takes advantage of the use of a standardized meta-analysis method and provided access to data on a large number of adult subjects of both genders and with a wide range of birth weight and ages [21].

## Methods

### Study Design

The present study is based on a Nordic longitudinal epidemiologic research program “Prenatal and Childhood Growth in Relation to Cardiovascular Disease” (the NordNet Study). All Nordic cohort studies that could provide information about birth weight and cardiovascular disease were invited to participate in the NordNet Study. The NordNet Study consists of researchers from 13 study centers located in Denmark, Finland, the Faroe Islands, Iceland, Norway and Sweden. They provided access to both published and unpublished raw data from 21 Nordic population-based studies of individuals constituting prospective and/or retrospective cohort studies followed longitudinally over many years and comprising >548.000 subjects in the collective data set. Out of the 13 participating centers, 10 centers [22]–[34] were able to contribute data on birth weight together with adolescent or adult LTPA and body mass index for a total of 13 cohorts. Twelve cohorts [22]–[28], [30]–[34] included 24,890 men (table 1), and 9 cohorts [22]–[24], [26]–[29], [31], [33], [34] included 18,592 women (table 2). Cohort participants were born between 1914 and 1987. All studies were performed in accordance with the Helsinki 2 Declaration. The meta-analysis was conducted on anonymous data, and it was approved by the Danish Data Protection Agency (*Datatilsynet*).

**Table 1:** Male cohorts included in the meta-analysis of birth weight and leisure time physical activity (LTPA).

Country and study	Birth- year(s)	Age (years)	Number of subjects	Mean birth weight (kg)	LTPA (% active)	Definition of LTPA (inactivity)
**Denmark**						
Andersen et al. (2000)	1932–1970	20–50	948	3.4 (0.56)^a^	79.1	None or very little
Bak et al. (2004)	1930–1956	26–52	225	3.5 (0.58)	58.7	<2 h of exercise/wk
Osler et al. (2008)	1953	51	5767	3.4 (0.53)	82.4	Read, watch television or have other sedentary activities
Willumsen (1970)	1959–1961	41–43	1856	3.3 (0.59)	54.0	Light physical activity <4 h/wk
Johnsen et al. (2006)	1932–1946	50–64	4143	3.4 (0.59)	74.4	1st quartile of MET-score for walking, cycling, sports, and gardening
**Finland**						
Salonen (1988)	1926–1946	42–61	719	3.5 (0.54)	54.1	Exercising < once/mo
Tammelin et al. (2003)	1966	31	2803	3.6 (0.52)	69.9	Brisk exercise <1 time/wk and light exercise <4 times/wk
Tammelin et al. (2007)	1986	15–16	2622	3.7 (0.51)	70.6	≤1 h/wk of brisk physical activity
Eriksson et al. (2006)	1934–1944	56–69	906	3.5 (0.50)	76.9	1st quartile of MET-score for conditioning and non-conditioning exercise
**Norway**						
Holmen et al. (2000)	1975–1981	14–20	2596	3.6 (0.54)	81.7	<1 time/wk and <2 h/wk of at least moderate intensity
**Sweden**						
Rasmussen et al. (2004)	1985–1987	14–16	1312	3.5 (0.54)	66.6	Lowest tertile of energy expenditure
Michealsson et al. (2007)	1920–1924	55–64	993	3.6 (0.50)	88.6	Mostly sedentary leisure time

a Numbers in parentheses, standard deviation.

**Table 2:** Female cohorts included in the meta-analysis of birth weight and leisure time physical activity (LTPA).

Country and study	Birth- year(s)	Age (years)	Number of subjects	Mean birth weight (kg)	LTPA (% active)	Definition of LTPA (inactivity)
**Denmark**						
Andersen et al. (2000)	1932–1970	20–50	919	3.3 (0.55)^a^	73.8	None or very little
Willumsen (1970)	1959–1961	41–43	2160	3.2 (0.56)	49.5	Light physical activity <4 h/wk
Johnsen et al. (2006)	1932–1946	50–64	4363	3.3 (0.59)	72.8	1st quartile of MET-score for walking, cycling, sports, and gardening
**Finland**						
Tammelin et al. (2003)	1966	31	2847	3.5 (0.49)	75.1	Brisk exercise <1 time/wk and light exercise <4 times/wk
Tammelin et al. (2007)	1986	15–16	2911	3.5 (0.51)	60.1	≤1 h/wk of brisk physical activity
Eriksson et al. (2006)	1934–1944	56–69	1059	3.4 (0.47)	73.5	1st quartile of MET-score for conditioning and non-conditioning exercise
**Norway**						
Holmen et al. (2000)	1975–1981	14–20	2512	3.5 (0.51)	79.3	<1 time/wk and <2 h/wk of at least moderate intensity
**Sweden**						
Rasmussen et al. (2004)	1985–1987	14–16	1214	3.4 (0.54)	66.7	Lowest tertile of energy expenditure
Lissner et al. (1996)	1914–1930	50–66	607	3.5 (0.54)	73.0	Almost completely inactive

a Numbers in parentheses, standard deviation.

Birth weight (in kg) was used as a categorical variable and divided into 11 categories: 0.75–1.25, 1.26–1.75, 1.76–2.25, 2.26–2.75, 2.76–3.25, 3.26–3.75, 3.76–4.25, 4.26–4.75, 4.76–5.25, 5.26–5.75, and 5.76–7.00 kg. The borders of the categories were chosen to minimize the arbitrary effect of digit preference. The birth weight category 3.26–3.75 kg was chosen as the reference category as it contained the most subjects. The birth weight groups 0.75–1.25, 5.26–5.75, and 5.76–7.00 kg were excluded in the analyses as they consisted of few subjects, are special cases, and are more likely subject to errors (table 3). Birth weight was either measured (10 cohorts) or reported by the mother (3 cohorts).

**Table 3:** Distribution of birth weight.

	Men		Women		All subjects
Birth weight; kg	14–34 y	35–74 y	14–34 y	35–74 y	14–74 y
0.75–1.25	6 (0.1)	9 (0.1)	13 (0.1)	19 (0.2)	47 (0.1)
1.26–1.75	21 (0.2)	73 (0.5)	31 (0.3)	69 (0.8)	194 (0.4)
1.76–2.25	83 (0.9)	327 (2.2)	108 (1.1)	257 (2.9)	775 (1.8)
2.26–2.75	372 (3.8)	1,107 (7.3)	541 (5.5)	951 (10.8)	2,971 (6.8)
2.76–3.25	1,873 (19.3)	4,093 (27.0)	2,443 (25.0)	2,883 (32.7)	11,292 (26.0)
3.26–3.75	3,664 (37.7)	5,592 (36.9)	3,987 (40.8)	3,113 (35.3)	16,356 (37.6)
3.76–4.25	2,727 (28.0)	3,077 (20.3)	2,127 (21.7)	1,195 (13.6)	9,126 (21.0)
4.26–4.75	848 (8.7)	741 (4.9)	471 (4.8)	247 (2.8)	2,307 (5.3)
4.76–5.25	117 (1.2)	120 (0.8)	57 (0.6)	53 (0.6)	347 (0.8)
5.26–5.75	14 (0.1)	12 (0.1)	4 (0.0)	12 (0.1)	42 (0.1)
5.76–7.00	2 (0.0)	12 (0.1)	1 (0.0)	10 (0.1)	25 (0.1)
**Total**	**9,727 (100)**	**15,163 (100)**	**9,783 (100)**	**8,809 (100)**	**43,482 (100)**

Number of subjects (%) for each birth weight group by sex and age and for all subjects.

Information on LTPA was obtained by questionnaire including information on duration, frequency, and/or intensity of the LTPA. Based on each cohort's information that consisted of either three to six levels of LTPA or of energy expenditure, we categorized LTPA as *active* or *inactive*. To construct comparable groups across cohorts, we defined the active category by a similar duration, frequency, and/or intensity of LTPA within age groups with the aim of having similar frequency of inactive subjects across cohorts. The categorizations were created in consultation with the investigators from each cohort.

The following potential confounders or mediators were investigated: gestational age (</>37 weeks), duration of education (as defined in each cohort), adult body mass index, and smoking (yes/no). Body mass index (weight/height^2^; kg/m^2^) was calculated for each subject based on self-reported (3 cohorts) or measured weight and height (10 cohorts) at the time of the physical activity assessment, and smoking habits were recorded on the same occasion.

### Statistical Methods

All analyses were performed in three steps. First, researchers responsible for each cohort performed the analyses described below stratified by sex and age; age was categorized as 14–17, 18–24, 25–34, 35–44, 45–54, 55–64, and 65–74 years. Second, the estimates of stratum-specific regression coefficients (SSRC), along with the corresponding standard errors, were reported to the coordinating center. Third, the estimated SSRCs were pooled using random-effects meta-analyses techniques with random effects based on strata formed from combinations of cohort-, sex-, and age-categories. Our procedure has been described in detail elsewhere [21].

We performed logistic regression of physical activity, modelling the probability of being active, on birth weight used as categorical variable for each combination of cohort and sex and age categories. We also used the SSRCs from the cohort and sex and age specific logistic regression analyses to investigate a potential heterogeneity of the association by age. In these analyses, the subjects were subdivided into two age groups consisting of a comparable number of subjects; a younger group aged 14–34 years and an older group aged 35–74 years. Moreover, we performed meta-regression with age as covariate to test for an age-effect, but there were no significant indications of this being the case. In cohorts where data on potential confounding or mediating factors were available, we performed logistic regressions of LTPA on birth weight used as a categorical variable both adjusted and unadjusted for the potential factors, one at a time, to assess their effects. To assess the impact of self-reported birth weight, we performed a sensitivity analysis omitting the 3 cohorts in which birth weight was self-reported.

All cohorts within the context of the NordNet collaboration that were eligible for this study were included irrespective of whether results on birth weight and LTPA had been published or not, and hence, the results of the present study should not be subject to publication bias. Given this analytic framework, our meta-regression analysis was not conducted in conjunction with a traditional systematic review of the literature. There are few published studies on this topic, and those that exist are difficult to compare due to different characteristics of subjects [4], [12], [17]–[20]. Thus, the method we used is advantageous over a standard meta-analysis and systematic literature review. If our access to data was biased by being dependent on the strength or direction of the birth weight-LTPA association, the cohorts included in our study could represent a biased pool of the theoretical sample. However, as inclusion of study cohorts in the Nordnet Study was based on availability of birth weight and cardiovascular disease information access bias is unlikely to have occurred.

## Results

**Figure 1:**
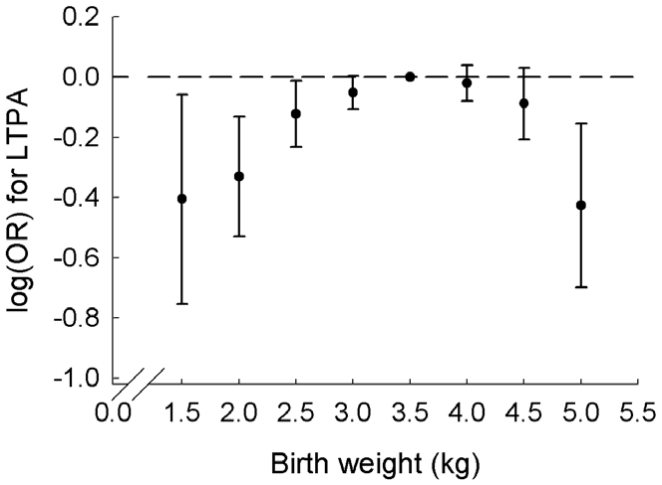
Meta-analysis of logistic regressions of leisure time physical activity as a function of birth weight. The points depict log(OR) and corresponding 95% confidence intervals with birth weight as a categorical variable. Only results for the birth weight groups in the interval 1.26–5.25 kg are presented. The dotted reference line corresponds to an OR value of 1.

Results from a meta-analysis, pooling all the cohort and age and sex specific regression coefficients from the logistic regression of birth weight as a categorical variable, revealed a reverse U-shaped association between birth weight and LTPA (figure 1). Lower probabilities of being active, when compared to the odds of engaging in LTPA among subjects in the reference category (3.26–3.75 kg; odds ratio (OR) of 1.00), were apparent at birth weights <2.76 kg and >4.75 kg, when examined for men and women combined. The ORs of engaging in LTPA for birth weights of 1.26–1.75, 1.76–2.25, 2.26–2.75, 4.26–4.75 and 4.76–5.25 kg were 0.67 (95% confidence interval (CI): 0.47, 0.94), 0.72 (95% CI: 0.59, 0.88), 0.89 (95% CI: 0.79, 0.99), 0.92 (95% CI: 0.81, 1.03), and 0.65 (95% CI: 0.50, 0.86), respectively. The strength of the effect of a low or a high birth weight on the probability of engaging in LTPA thus appeared to be similar. The birth weight groups 2.76–3.25 and 3.76–4.25 kg had only slightly lower probabilities of undertaking LTPA than the reference group. Thus, there was no noteworthy birth weight-LTPA association in the main part of the normal birth weight interval. When omitting the 3 three cohorts in which birth weight was self-reported, the reverse U-shaped association remained, although it was slightly attenuated and became non-significant (data not shown).

Analyses of birth weight as a categorical variable conducted separately for men and women showed no sex-dependency in the birth weight-LTPA association (table 4). The reverse U-shaped association between birth weight and the odds of engaging in LTPA was apparent among both men and women and the strength of the reverse U-shaped birth weight-LTPA association was similar in men and women.

**Table 4:** Logistic regression of leisure time physical activity on birth weight.

	Men		Women	
Birth weight; kg^a^	14–34 y	35–74 y	14–34 y	35–74 y
1.76–2.25	0.82 [0.50, 1.36]	0.77 [0.60, 0.98]	0.73 [0.46, 1.15]	0.64 [0.40, 1.01]
2.26–2.75	1.12 [0.88, 1.44]	0.79 [0.65, 0.95]	0.89 [0.73, 1.09]	0.92 [0.79, 1.08]
2.76–3.25	1.08 [0.95, 1.22]	0.87 [0.79, 0.96]	0.99 [0.89, 1.11]	0.93 [0.83, 1.04]
3.26–3.75	1.0 (ref)	1.0 (ref)	1.0 (ref)	1.0 (ref)
3.76–4.25	0.97 [0.87, 1.09]	0.92 [0.83, 1.03]	1.05 [0.93, 1.18]	1.01 [0.87, 1.17]
4.26–4.75	0.98 [0.83, 1.17]	0.79 [0.62, 1.00]	1.01 [0.81, 1.26]	0.85 [0.64, 1.13]
4.76–5.25	0.73 [0.50, 1.10]	0.41 [0.27, 0.65]	1.01 [0.43, 2.36]	0.78 [0.42, 1.47]

Odds ratio [95% confidence limits] for each birth weight category among men <35 years and ≥35 years and among women <35 years and ≥35 years.

a Results from the birth weight groups 0.75–1.25, 1.26–1.75, 5.26–5.75, and 5.76–7.0 kg were excluded because of lack of available data.

The results from the regression analyses of LTPA on birth weight treated as a categorical variable separately for men and women younger than or at least 35 years showed limited evidence of an age modification of the shape or strength of the birth weight-LTPA association (table 4).

The effect of potential confounders or mediators was investigated separately for men and women. The results are not shown, but are provided in Table S1 and Table S2 in supplementary online material. Including gestational age in the model appeared to reduce the OR of LTPA among low and high birth weight men and women slightly further, indicating a stronger effect of birth weight on LTPA when the effect of gestational age was taken into account. The estimates, however, were less precise because 5 cohorts did not have the information available. Comparing the birth weight-LTPA association estimates unadjusted and adjusted for education, body mass index, and smoking status, one at a time, showed essentially no changes in the odds of undertaking LTPA and thus no indication of confounding or mediation effects.

## Discussion

This study investigated birth weight as a determinant of a behavioral outcome in terms of physical activity during leisure time among adolescents and adults. The study indicated that both very low and very high birth weights were associated with a lower probability of undertaking LTPA, whereas the association between birth weight and LTPA was weak in the birth weight interval of 2.76–4.25 kg, which is the range that contains the majority of subjects. These findings were obtained irrespective of adjustment for potential confounders or mediators, although unmeasured or residual confounding cannot be excluded.

A few other studies have investigated the association between size at birth and physical activity in adulthood. In 462 middle-aged Finnish men, ponderal index at birth was not associated with the duration of strenuous leisure time physical activity [4]. The shape of the association cannot be evaluated since only the p-value for the association is reported. In a cohort of 186 elderly Finnish men, birth weight and ponderal index were, in contrast to the findings of the present study, significantly inversely correlated with exercise frequency. These analyses were adjusted for age and current body mass index, but not for gestational age. The associations were not significant in the 314 women included in this cohort [19]. Conversely, 163 very-low-birth-weight (≤1500 g) young Finnish adults were active at a lower intensity while undertaking regular leisure-time exercise compared to 169 subjects born at term [20]. Similarly, a much larger study of 25,874 British middle-aged adults showed that a higher birth weight, expressed continuously and linearly, was significantly and positively associated with the prevalence of undertaking regular physical activity [18].

Fewer studies have investigated physical activity in subjects with a high birth weight. The British study included subjects with a high birth weight, and according to the crude prevalence rates of undertaking regular physical activity, those with a birth weight >4.5 kg were less likely to undertake regular physical activity than subjects with a birth weight between 4.0 and 4.5 kg [18].

Physical activity is a multidimensional and complex exposure to measure. The studies of size at birth and physical activity have investigated different aspects of physical activity, and this could contribute to the differing results. The studies were conducted in age groups from young adults to the elderly, but we found, however, limited evidence of an age modification of the birth weight-LTPA association.

Although the other studies did not explicitly investigate the shape of the association between birth weight and occurrence of LTPA later in life throughout the entire birth weight range, the association seems to be an inverse U-shape and very weak within the normal birth weight range.

The underlying mechanisms for the present findings are unknown, but there are several possible explanations. Low birth weight could be associated with a reduced physical capacity through a variety of biological mechanisms, including a reduced muscle strength because of low muscle mass [15], [16], [35], [36] that can also be measured as reduced hand grip strength [15], [37], [38]. Moreover, it could work through an insufficient anaerobic capacity [39], respiratory capacity [40], aerobic fitness [41], or even a higher energetic cost of exercise [42]. These biological factors could reduce the willingness to undertake physical activity because of early tiredness and a reduced ability to perform physical activity. Among adults, a high birth weight has been associated with an increased risk of obesity as indicated by high body mass index in later life [7]–[10]. This biological factor could reduce the aerobic capacity or reduce the motivation for physical activity because of discomfort as previously suggested [26], [43] and thereby lead to a lower level of physical activity among subjects with a high birth weight.

We did not find any obvious confounding or mediating effect of indicators of concurrent social position, body mass index, or smoking status on the birth weight-LTPA association. We cannot, however, exclude the possibility that unmeasured or residual confounding, for example from genes or aspects of social background, may contribute to explain our findings. Gestational age could be regarded as a determinant of birth weight, and adjusting for gestational age strengthened the birth weight-LTPA association indicating that the intrauterine growth pattern, i.e. birth weight for gestational age, may be a stronger determinant for LTPA than the birth weight per se. This finding is in accordance with studies on birth weight and coronary heart disease, which found that the association was strengthened by adjustment for gestational age [44], [45].

In the NordNet collaboration, we used a standardized meta-regression method to investigate the association between birth weight and LTPA. Researchers conducting meta-analyses are often limited to published estimates that may not be very consistent with the question of interest. In our study we developed and applied a common analytic strategy. For example, we were able to create a consistent as possible definition of LTPA and to specify which potential confounding variables to adjust for in every cohort, thus allowing us to obtain uniform estimates from each cohort. As with any meta-analysis, there was an assumption that the included cohorts were homogeneous, and these methods helped reduce any impact of non-homogeneity. Additionally, the use of a random effects meta-analysis model allowed any potential heterogeneity in the association between birth weight and LTPA across cohorts to occur around a normally distributed mean effect. The NordNet collaboration circumvented the potential of publication biases usually encountered in meta-analysis on previously published results, as many results that may have had difficulties being published on their own because of null-findings were included in this study. Moreover, this set-up overcame problems experienced in traditional meta-analyses that are unable to adjust for confounding, and allowed for the discovery of the inverse U-shaped birth weight-LTPA association, which would not have been revealed by a simple linear regression.

Some information on birth weight and all information on physical activity was self-reported, and thus has an inherent potential for diminished precision. However, a sensitivity analysis showed no influence of self-reported birth weight on the results. There is always a potential for misclassification bias in self-reported estimation of LTPA. However, most likely, any misclassification of LTPA will attenuate the strength of the observed association. Moreover, although simple questionnaires give a relatively crude measure of overall physical activity, they are useful in larger cohort studies [46]. The physical activity assessed in the present study may thus represent active or inactive lifestyle patterns rather than exact levels of physical activity justifying the comparison of active and inactive within each cohort and the pooling of the results.

This study suggested that factors in early life might influence physical activity behavior in adult life. The results of this study do not necessarily imply recommendations for interventions to modify birth weight; birth weight is a proxy for many determinants, which have not yet fully been identified, and, moreover, birth weight is not easily modifiable [47]. The lower probability of engaging in LTPA observed among subjects with birth weights lower than the reference groups outside the normal range of birth weight may have implications for public health. If physical activity constitutes a link between birth weight and morbidity and mortality, promotion of physical activity may be of special importance among subjects of low and high birth weights.

In summary, this study provides evidence that both low and high birth weights outside the normal range of birth weights are associated with a lower probability of being physically active during leisure time. This may be part of the underlying mechanism of the association between birth weight and disease and may thus have public health implications.

## Supporting Information

Table S1 Logistic regression of leisure time physical activity on birth weight with adjustments among men.
(0.01 MB PDF)

Table S2 Logistic regression of leisure time physical activity on birth weight with adjustments among women.
(0.01 MB PDF)
