# Supplementary material for: Birth Weight in Relation to Leisure Time Physical Activity in Adolescence and Adulthood: Meta-Analysis of Results from 13 Nordic Cohorts
Source: PLoS One. 2009 Dec 16;4(12):e8192. doi: 10.1371/journal.pone.0008192 (PMC2790716; doi:10.1371/journal.pone.0008192)
Supplement: Table S1 — Logistic regression of leisure time physical activity on birth weight with adjustments among men. (0.01 MB PDF) [file pone.0008192.s001.pdf]

Birth weight; kg	Unadjusted[1]	Adjusted for gestational age[1]	Unadjusted[1]	Adjusted for education[1]	Unadjusted[2]	Adjusted for body mass index[2]	Unadjusted[3]	Adjusted for smoking[3]
**1.26-1.75**	0.90 [0.45, 1.79]	0.86 [0.42, 1.77]	NA	NA	0.77 [0.49, 1.19]	0.80 [0.50, 1.29]	0.75 [0.37, 1.52]	0.72 [0.37, 1.40]
**1.76-2.25**	0.86 [0.59, 1.25]	0.86 [0.54, 1.39]	0.77 [0.61, 0.98]	0.82 [0.64, 1.04]	0.78 [0.62, 0.97]	0.87 [0.66, 1.15]	0.75 [0.59, 0.95]	0.77 [0.60, 0.98]
**2.26-2.75**	1.01 [0.79, 1.28]	0.85 [0.63, 1.16]	0.83 [0.68, 1.00]	0.82 [0.69, 0.99]	0.89 [0.75, 1.07]	0.89 [0.72, 1.10]	0.92 [0.76, 1.11]	0.91 [0.76, 1.10]
**2.76-3.25**	1.06 [0.94, 1.18]	1.07 [0.88, 1.31]	0.90 [0.82, 0.98]	0.90 [0.81, 1.00]	0.95 [0.87, 1.03]	0.91 [0.76, 1.09]	0.95 [0.87, 1.03]	0.96 [0.88, 1.05]
**3.26-3.75**	1.0 (ref)	1.0 (ref)	1.0 (ref)	1.0 (ref)	1.0 (ref)	1.0 (ref)	1.0 (ref)	1.0 (ref)
**3.76-4.25**	0.95 [0.86, 1.05]	0.90 [0.73, 1.12]	0.95 [0.87, 1.05]	0.92 [0.82, 1.03]	0.95 [0.87, 1.02]	0.91 [0.76, 1.10]	0.96 [0.88, 1.04]	0.96 [0.88, 1.05]
**4.26-4.75**	0.89 [0.76, 1.04]	0.71 [0.53, 0.95]	0.87 [0.74, 1.04]	0.83 [0.67, 1.04]	0.91 [0.80, 1.03]	0.86 [0.69, 1.09]	0.91 [0.79, 1.04]	0.90 [0.79, 1.04]
**4.76-5.25**	0.72 [0.48, 1.06]	0.61 [0.29, 1.26]	0.49 [0.33, 0.73]	0.48 [0.32, 0.72]	0.57 [0.42, 0.77]	0.54 [0.37, 0.79]	0.56 [0.40, 0.79]	0.55 [0.39, 0.79]

Odds ratio [95% confidence limits] for each birth weight category.

[1] Based on 10 cohorts.

[2] Based on all 13 cohorts.

[3] Based on 12 cohorts.
