# Supplementary material for: Birth Weight in Relation to Leisure Time Physical Activity in Adolescence and Adulthood: Meta-Analysis of Results from 13 Nordic Cohorts
Source: PLoS One. 2009 Dec 16;4(12):e8192. doi: 10.1371/journal.pone.0008192 (PMC2790716; doi:10.1371/journal.pone.0008192)
Supplement: Table S2 — Logistic regression of leisure time physical activity on birth weight with adjustments among women. (0.01 MB PDF) [file pone.0008192.s002.pdf]

Birth weight; kg	Unadjusted[1]	Adjusted for gestational age[1]	Unadjusted[1]	Adjusted for education[1]	Unadjusted[2]	Adjusted for body mass index[2]	Unadjusted[3]	Adjusted for smoking[3]
**1.26-1.75**	0.92 [0.47, 1.78]	0.90 [0.46, 1.74]	0.57 [0.36, 0.91]	0.61 [0.38, 0.97]	0.61 [0.39, 0.95]	0.68 [0.42, 1.10]	0.59 [0.34, 1.02]	0.59 [0.36, 0.95]
**1.76-2.25**	0.32 [0.04, 2.26]	0.24 [0.02, 2.50]	0.58 [0.37, 0.91]	0.60 [0.38, 0.95]	0.70 [0.50, 0.99]	0.76 [0.56, 1.02]	0.68 [0.45, 1.01]	0.69 [0.45, 1.06]
**2.26-2.75**	0.91 [0.78, 1.07]	0.91 [0.71, 1.16]	0.95 [0.82, 1.09]	0.93 [0.80, 1.09]	0.91 [0.80, 1.03]	0.93 [0.74, 1.18]	0.96 [0.83, 1.10]	0.95 [0.82, 1.10]
**2.76-3.25**	0.97 [0.88, 1.07]	0.90 [0.75, 1.08]	0.93 [0.85, 1.03]	0.93 [0.83, 1.05]	0.96 [0.89, 1.04]	0.95 [0.77, 1.18]	0.96 [0.88, 1.05]	0.95 [0.87, 1.05]
**3.26-3.75**	1.0 (ref)	1.0 (ref)	1.0 (ref)	1.0 (ref)	1.0 (ref)	1.0 (ref)	1.0 (ref)	1.0 (ref)
**3.76-4.25**	1.01 [0.91, 1.12]	0.87 [0.70, 1.08]	1.03 [0.91, 1.17]	1.03 [0.88, 1.19]	1.03 [0.94, 1.13]	1.04 [0.83, 1.31]	1.04 [0.94, 1.15]	1.04 [0.93, 1.15]
**4.26-4.75**	1.06 [0.86, 1.31]	1.17 [0.78, 1.78]	0.84 [0.67, 1.07]	0.84 [0.65, 1.08]	0.95 [0.79, 1.13]	0.90 [0.68, 1.19]	0.96 [0.79, 1.16]	0.92 [0.76, 1.11]
**4.76-5.25**	1.01 [0.43, 2.38]	0.49 [0.14, 1.67]	0.93 [0.52, 1.65]	0.95 [0.53, 1.71]	0.91 [0.57, 1.43]	0.95 [0.56, 1.61]	0.98 [0.51, 1.90]	0.98 [0.50, 1.93]

Odds ratio [95% confidence limits] for each birth weight category.
[1] Based on 10 cohorts.
[2] Based on all 13 cohorts.
[3] Based on 12 cohorts.
